# Superior Safety and Surgical Efficiency by Automated Vacuum-Assisted Venous Drainage Over Conventional, Survey-Based Evaluation

**DOI:** 10.1093/icvts/ivaf165

**Published:** 2025-07-22

**Authors:** Youssef El Dsouki, Ignazio Condello

**Affiliations:** Cardiovascular and Clinical Perfusion Department, Universitè, Paris-Sorbonne, 75005 Paris, France; School of Medicine and Surgery, University of Insubria, 21100 Varese, Italy

**Keywords:** automated, conventional, manual, vacuum-assisted venous drainage, cardiopulmonary bypass, extracorporeal circulation, survey

## Abstract

The evolution of cardiopulmonary bypass (CPB) techniques has been significantly enhanced by the integration of vacuum-assisted venous drainage (VAVD). Vacuum-assisted venous drainage improves venous return in minimally invasive, paediatric, and complex cardiac surgeries by reducing priming volumes and minimizing hemodilution. However, excessive negative pressure can lead to risks, such as air embolism, hemolysis, and circuit collapse. This study aimed to compare automated and manual (conventional) VAVD systems regarding key safety and performance parameters: (1) pressure stability under varying venous return conditions, (2) incidence of air embolism and vacuum-related complications, (3) alarm response times, and (4) hemolysis. A survey was conducted across 23 cardiac surgery centres, although 3 centres did not specify the type of VAVD device used and were excluded from data analysis. The final analysis included 20 centres: 10 using automated systems and 10 using conventional systems. Data were collected using a standardized checklist assessing real-time vacuum control, safety alarms, and complication rates. Results demonstrated that automated VAVD systems provided more consistent pressure stability (98%), reduced hemolysis rates (<3%), improved alarm response times (<2 seconds), and fewer air embolism events compared to manual systems.

## BACKGROUND

The evolution of cardiopulmonary bypass (CPB) techniques has been significantly enhanced by integrating vacuum-assisted venous drainage (VAVD). Vacuum-assisted venous drainage has become an essential tool in modern CPB, improving venous return in minimally invasive, paediatric cardiac surgery, and complex cardiac surgeries. Where precision and control over venous drainage are crucial due to the small blood volumes and delicate physiological conditions. Vacuum-assisted venous drainage allows for the use of smaller cannulas and shorter circuit lengths, significantly reducing priming volumes, thus minimizing hemodilution and the need for blood transfusions.^1^^,^^2^ This consistency in venous return not only improves surgical field visibility but also reduces physiological stress on patients, potentially leading to quicker recoveries and fewer postoperative complications.^3^ However, excessive negative pressure can lead to risks, such as increase of air embolism, hemolysis, and circuit collapse.^4^^,^^5^ This study is based on the hypothesis that automated VAVD systems are superior to manual VAVD systems in terms of safety, consistency, and overall clinical performance during CPB. The objective is to evaluate this hypothesis through a comparative analysis of key parameters, including pressure stability, incidence of air embolism, alarm response times, and hemolysis.

## MATERIALS AND METHODS

This was a cross-sectional survey study conducted from January to March 2025, defined as the collection of information from a sample of individuals through their responses to a standardized questionnaire. The survey aimed to assess the comparative performance and safety of automated versus conventional VAVD systems in cardiac surgery centres. Data were collected in the form of a standardized survey using SurveyMonkey, an online platform that allowed for anonymous and structured data collection. The questionnaire consisted of both closed-ended and structured response items covering technical and clinical observations regarding the use of VAVD systems. Given that this study involved a survey of anonymized technical data regarding perfusionist users of VAVD systems, Institutional Review Board (IRB) approval and informed consent were not required.

The survey included the following domains:

- type of VAVD system used (automated, manual, unspecified);
- pressure stability under varying venous return conditions;
- incidence of air embolism;
- frequency and responsiveness of alarm systems;
- occurrence of hemolysis;
- need for operator intervention; and
- system reliability and ease of use.

Participants were perfusionists and cardiac surgery team members involved in the intraoperative management of CPB. Demographic data were not collected in this version of the study. The primary outcomes of the study were pressure stability, incidence of air embolism, alarm response time, and hemolysis rates. Secondary outcomes included system usability and the frequency of manual interventions required. Statistical analyses were descriptive in nature, including frequency counts, percentages, and comparative analysis between centres using automated versus conventional VAVD systems. Given the exploratory nature and limited sample size, no inferential statistical testing was applied. Centres were included if they provided complete information regarding the type of VAVD system in use. Three centres were excluded from the analysis as they did not specify whether the system used was automated or conventional.

A flow chart has been included to illustrate the survey response pathway and exclusion criteria:

- total surveys distributed: 23;
- total responses received: 23;
- surveys excluded due to unspecified device: 3; and
- final surveys included in analysis: 20 (10 automated, 10 conventional).

**Figure 1.**
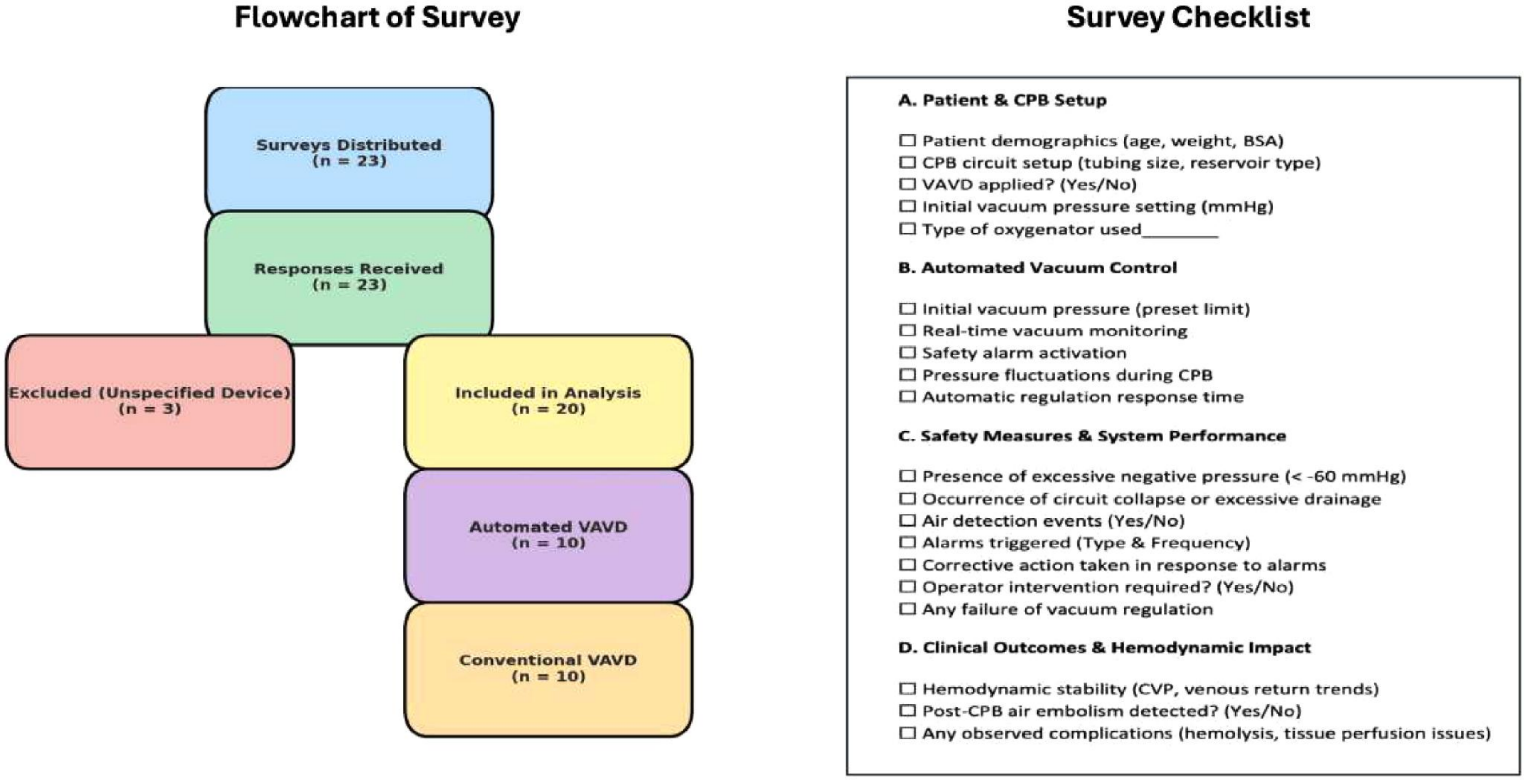
Flowchart and Checklist Used for Survey

Data were collected using a standardized assessment checklist, ensuring consistency across cases (**Figure 1**).

## RESULTS

A total of 23 adult cardiac surgery centres completed the survey. Three centres were excluded from the final analysis due to unspecified VAVD device usage, resulting in a final sample of 20 centres—equally divided between automated and conventional VAVD systems (*n* = 10 per group). Across the analysed parameters, automated VAVD systems demonstrated superior performance compared to conventional systems. Most notably, pressure stability was consistently reported in nearly all automated centres, while a decline in consistency was noted among conventional users. Safety indicators, such as hemolysis rates and air-related complications, also favoured automated systems, which reported minimal occurrences (<3% and <1%, respectively). Conventional systems, by contrast, showed hemolysis incidence up to 10% and air-related events as high as 8%. Similarly, post-CPB air embolism was nearly absent in the automated group but present in 7% of conventional cases. In terms of response efficiency, automated systems triggered alarms in under 2 seconds, whereas response times in conventional systems ranged from 6 to 10 seconds. These differences were echoed in the reported occurrence of excessive negative pressure and drainage, both of which were markedly lower in the automated group. Finally, subjective assessments of system reliability were more favourable for automated systems, with 90% of respondents rating them positively, compared to only 60% for conventional systems. **Table 1** provides a detailed comparison of all surveyed parameters between the 2 groups.

**Table 1.** Comparison of Key Perfusion Parameters Between Automated and Conventional VAVD Systems in Adult Cardiac Surgery Centres

Parameter	Automated VAVD (*n* = 10)	Conventional VAVD (*n* = 10)
Pressure stability	98%	80%
Hemolysis incidence	<3%	10%
Alarm response time	<2 seconds	6-10 seconds
Excessive negative pressure	<5%	20%
Excessive drainage	1%	12%
Air-related complications	<1%	8%
Post-CPB air embolism	<1%	7%
System reliability rating (subjective)	High (90% positive)	Moderate (60% positive)

Abbreviation: CPB, cardiopulmonary bypass; VAVD, vacuum-assisted venous drainage.

## DISCUSSION

Although comprehensive global data on the prevalence of automated versus conventional VAVD systems remain limited, insights from our survey of 23 cardiac surgery centres in the Middle East offer a preliminary view of current practices in the region. In our sample, approximately 43% (10 centres) reported the use of automated VAVD systems, 43% (10 centres) used conventional systems, and 13% (3 centres) did not specify the type employed. While this sample size is limited and not necessarily representative of the entire region, it does suggest a gradual shift towards automated technologies in perfusion practice. This trend may reflect growing interest in improving safety, standardization, and procedural efficiency in cardiac surgery. The effectiveness of manual VAVD heavily relies on the skill and continuous attention of the operator. This dependency can lead to variability in patient care and outcomes.^6^^,^^7^ The need for continuous manual adjustments introduces a high risk of human error, particularly under the stress of critical phases of surgery. This can lead to inappropriate vacuum levels, which might result in mechanical trauma or other complications.^8^^,^^9^ Manual adjustments require significant cognitive load from the surgical team, potentially causing distractions during critical phases of the operation. This not only affects the efficiency of the procedure but may also compromise patient safety.^10^

### Study limitations

This study has several limitations that should be considered when interpreting the results. First, as a survey-based analysis, it is subject to inherent limitations, such as sampling bias, recall bias, and potential inconsistencies in self-reported data. The survey relied on voluntary participation, which may have introduced selection bias, as centres with strong opinions or experiences (positive or negative) regarding VAVD systems may have been more likely to respond. Additionally, the sample was limited to 23 centres within a specific region (Middle East), which may not fully reflect global practices or outcomes. Another important limitation is the absence of demographic and institutional characteristics, such as the size of the centres or experience level of the perfusion staff, which could influence perceptions and outcomes related to VAVD use. Future iterations may include role-specific or institutional demographic information to allow for subgroup analysis. Furthermore, we did not employ validated psychometric testing for the survey instrument, which may limit the measurement validity and reliability of certain subjective responses. Future studies with larger datasets will incorporate inferential statistics as recommended.

## CONCLUSION

The adoption of automated VAVD systems marks a pivotal advancement in CPB technology. These systems address critical limitations inherent in manual VAVD, including human error, inconsistencies in venous return management, and the risks of complications, such as hemolysis. By ensuring precise and consistent control of vacuum pressures, automated VAVD significantly reduces the potential for blood cell damage and enhances the overall safety and efficiency of CPB procedures. Comprehensive empirical studies are required to validate the long-term benefits, assess cost-effectiveness, and determine the adaptability of automated VAVD across various surgical environments and patient populations.

## Data Availability

All data supporting the findings of this study are available from the corresponding author upon reasonable request.

## References

[1] Berryessa R , Wiencek R, Jacobson J, et al Vacuum-assisted venous return in pediatric cardiopulmonary bypass. Perfusion. 2000;15:63-67. 10.1177/026765910001500109 10676869

[2] Vida VL , Bhattarai A, Speggiorin S, et al Effect of vacuum on venous drainage: an experimental evaluation on pediatric venous cannulas and tubing systems. JNMA J Nepal Med Assoc. 2014;52:960-966. 26982892

[3] Wang S , Undar A. Vacuum-assisted venous drainage and gaseous microemboli in cardiopulmonary bypass. J Extra Corpor Technol. 2008;40:249-256. 19192754 PMC4680714

[4] Saczkowski R , Zulauf F, Spada S. An evaluation of hard-shell venous reservoir integrated pressure relief valve pressure mitigation performance. Perfusion. 2022;37:37-45. 10.1177/0267659120976278 33245009

[5] Davila RM , Rawles T, Mack MJ. Venoarterial air embolus: a complication of vacuum-assisted venous drainage. Ann Thorac Surg. 2001;71:1369-1371. 10.1016/s0003-4975(00)02198-6 11308201

[6] Kiyama H , Imazeki T, Katayama Y, et al Vacuum-assisted venous drainage in single-access minimally invasive cardiac surgery. J Artif Organs. 2003;6:20-24. 10.1007/s100470300003 14598120

[7] Durandy Y. The impact of vacuum-assisted venous drainage and miniaturized bypass circuits on blood transfusion in pediatric cardiac surgery. ASAIO J. 2009;55:117-120. 10.1097/MAT.0b013e31819142f1 19092654

[8] De Somer F. Venous drainage—gravity or assisted? Perfusion. 2011;26 Suppl 1:15-19. 10.1177/0267659110394713 21933817

[9] Wahba A , Kunst G, De Somer F, et al 2024 EACTS/EACTAIC/EBCP Guidelines on cardiopulmonary bypass in adult cardiac surgery. Br J Anaesth. 2025;134:917-1008. 10.1016/j.bja.2025.01.015 39955230 PMC11947607

[10] Carvalho Filho EB , Marson FA, Costa LN, et al Vacuum-assisted drainage in cardiopulmonary bypass: advantages and disadvantages. Rev Bras Cir Cardiovasc. 2014;29:266-271. 10.5935/1678-9741.20140029 25140478 PMC4389465

